# OxyGene: an innovative platform for investigating oxidative-response genes in whole prokaryotic genomes

**DOI:** 10.1186/1471-2164-9-637

**Published:** 2008-12-31

**Authors:** David Thybert, Stéphane Avner, Céline Lucchetti-Miganeh, Angélique Chéron, Frédérique Barloy-Hubler

**Affiliations:** 1 CNRS UMR 6026, Interactions Cellulaires et Moléculaires, Equipe B@SIC, Université de Rennes 1, IFR140 GFAS, Campus de Beaulieu, Av. du Général Leclerc, 35042 Rennes, France

## Abstract

**Background:**

Oxidative stress is a common stress encountered by living organisms and is due to an imbalance between intracellular reactive oxygen and nitrogen species (ROS, RNS) and cellular antioxidant defence. To defend themselves against ROS/RNS, bacteria possess a subsystem of detoxification enzymes, which are classified with regard to their substrates. To identify such enzymes in prokaryotic genomes, different approaches based on similarity, enzyme profiles or patterns exist. Unfortunately, several problems persist in the annotation, classification and naming of these enzymes due mainly to some erroneous entries in databases, mistake propagation, absence of updating and disparity in function description.

**Description:**

In order to improve the current annotation of oxidative stress subsystems, an innovative platform named OxyGene has been developed. It integrates an original database called OxyDB, holding thoroughly tested anchor-based signatures associated to subfamilies of oxidative stress enzymes, and a new anchor-driven annotator, for *ab initio *detection of ROS/RNS response genes. All complete Bacterial and Archaeal genomes have been re-annotated, and the results stored in the OxyGene repository can be interrogated via a Graphical User Interface.

**Conclusion:**

OxyGene enables the exploration and comparative analysis of enzymes belonging to 37 detoxification subclasses in 664 microbial genomes. It proposes a new classification that improves both the ontology and the annotation of the detoxification subsystems in prokaryotic whole genomes, while discovering new ORFs and attributing precise function to hypothetical annotated proteins. OxyGene is freely available at:

## Background

Oxidative stress is a key stress in bacteria, caused by an imbalance between intracellular oxidant concentration, cellular antioxidant defence and oxidative alteration of macromolecules (membrane lipids, proteins and DNA repair enzymes) [1]. The reactive oxygen species (ROS) and nitrogen species (RNS) are the principal causes of oxidative stress [2]. They are mainly constituted of the hydroxyl radical (^•^OH), the superoxide anion (O_2_^-^), hydrogen peroxide (H_2_O_2_), organic hydroperoxide (ROOH), peroxynitrite (OONO) and nitric oxide (NO). ROS and RNS cause damages to proteins [3-5], DNA molecules [6,7], RNA and lipids leading to dysfunctions of the cellular metabolism [8]. This toxicity of ROS/RNS reveals the importance of efficient protection subsystems, such as the detoxification subsystem that gathers enzymes classified with regard to their substrates. Catalases are universal enzymes found in nearly all-living organisms that degrade hydrogen peroxide to produce oxygen and water [9-11]. Peroxidases reduce hydrogen or organic peroxides into water and alcohol moiety. This class of enzymes encompasses a large number of phylogenetically unrelated families such as peroxiredoxins [12,13], rubrerythrins [14,15], glutathione-peroxidases [16] or haloperoxidases [17,18]. Superoxide dismutases (SOD) dismute superoxide into hydrogen peroxide and oxygen [19-21]. An additional mechanism recently described involves non-heme iron proteins called superoxide reductases (SOR) [22]. The latter catalyzes the one-electron reduction of superoxide into hydrogen peroxide. Finally, RNS-scavenging enzymes are essentially globins [23,24] and nitric oxide reductases [25,26].

The increasing number of sequenced prokaryotic genomes makes it possible to perform comparative genomic analyses, in order to gain insight in the evolutionary or functional processes of the detoxification subsystem. The fundamental step lies in the identification of the potentialities of the genome by searching all proteins implied in this subsystem. Bioinformatic identifications of genes in a genome are mostly performed by similarity searches (using tools like FASTA[27] or BLAST[28]) against the full non-redundant protein UniProt databank [29]. Additional tools have also been used to detect patterns (PROSITE [30,31], BLOCKS [32], SMART [33], PRODOM and CDD [34]) or structures (SCOP, [35,36]), to classify enzymes (PRIAM [37]) and to assign function (HAMAP [38]). Unfortunately, several problems persist in the annotation, classification and naming of these enzymes. Inconsistent gene function naming can result from erroneous annotation of closest homolog proteins in database entries. Classification of proteins of the same enzymatic class (*i.e*. catalase) but belonging to different sub-classes (haem-dependent monofunctional, bifunctional, Mn-dependent, etc.) is difficult using BLAST [28] and/or FASTA [27] analysis because all these sequences show significant amino acid similarities around their catalytic residue. Additionally, many unrelated functional sequences appear to have "significant" similarities [39].

To improve the annotation of ROS/RNS response subsystems and to bypass previous inaccurate computer-assisted annotations, we have developed a platform named OxyGene and an embedded supervised database (OxyDB) with a new ontology and unambiguous anchor-based signatures for 37 ROS/RNS detoxification enzymes. The package is freely available. Here, we describe the design of OxyGene, and the procedures used to develop the OxyDB database and validate the *ab initio *annotations. We also present the user-friendly OxyGene interface that facilitates browsing, visualization, downloads and comparisons of OxyGene *ab initio*-annotated detoxification subsystems in the entirely sequenced genomes of 612 Bacteria and 52 Archaea. We illustrate some of the uses of OxyGene, consider the resulting biological insights emerging from its use and describe possible future developments.

## Construction and content

### OxyGene annotation operating principles

#### Annotation by "subsystem"

OxyGene annotates sets of genes that implement the same oxidative stress response processes such as detoxification or reduction. Each set is called a "subsystem", following the definition developed by Overbeek *et al*. for the SEED annotation environment [40]. Thus a subsystem is an assembly of molecular functions that perform the same biological process, based on new controlled vocabularies and functional relationships. Each subsystem is assembled by a group of expert curators after mining all available (gene and protein) function assertions resources (including the literature, databases, and sequence similarity searches). Compared to SEED, the detoxification subsystem defined in OxyGene is more exhaustive than the SEED subsystem « Protection from Reactive Oxygen Species », which includes 6 functional roles (SodA, SodB, SodC, HPII, HPI, CCP) while OxyGene proposes 37 functional roles. Moreover, the level of details of each protein family in OxyGene has been refined by a phylogenetic tree approach to give a more precise classification than the one found in SEED.

#### *Ab initio *annotation

OxyGene performs an *ab initio *computational identification and classification of oxidative stress response genes, as most existing annotation outputs are unsuitable for data mining: this *de novo *annotation allowed (1) new *loci *to be detected, (2) genes to be relocated in terms of the coding frame or start codon, (3) new function descriptions to be proposed for previously annotated but hypothetical genes, (4) generic annotations (such a "oxidase") to be reformulated, and (5) existing inaccurate functional assertions to be detected.

#### Comprehensive and non-overlapping classification

Members of a protein class have the same general function (*e.g*. catalase or nitric oxide dioxygenase) but often include one or more subclasses with slightly different properties, such as substrate specificity. To annotate these functional differences, each protein class is divided into subclasses by manually inspecting and subdividing phylogenetic trees. Subdivision criteria are the distance, domain architecture (number of domains, size and fusion events) and data from the literature for each protein cluster. In OxyGene, subclasses are identified by OxyDB_IDs (*e.g*. OXY.1.1.1.-) that include OxyDB_Tags (*e.g*. CAT_MON), description (*e.g*. catalase monofunctional) and additional information (see OxyDB database section). See additional file 1 for an example of the classification provided for the catalase class and subclasses.

#### Annotation using "anchors"

The most common approach to associating a gene unambiguously with a function is "inheritance through homology", estimated using tools like BLAST [28], PSI-BLAST [28], or HMMER [41]. Although these tools have become ubiquitous for annotation, they suffer various limitations: there is no universal e-value cut-off criterion and the outputs can be skewed by the length of sequences. Moreover, we found that these tools were unable to differentiate closely related subclasses: using the CAT_SRP catalase subclass as an input (for both BLAST and PSI-BLAST tools), other subclasses (CAT_MON and CAT_GAT) were also recruited with highly significant and overlapping e-values (illustrated in additional file 2). The HMMER approach gave better results for the catalase family as each profile specifically recruits each subclass (data not shown). However, the efficiency of HMMER depends on the dataset because, in profile-based approaches, all positions of the sequence alignment influence the final score. This influence may prevent precise discrimination between two closely related subfamilies, especially when the sequences are short and the number of specific positions small. This is the case for instance of the truncated-globin subclasses, wherein the GLB_TRO profile recruits GLB_TRP sequences (additional file 2). Because the anchor-based approach is strict, it unambiguously discriminates sequences that have different specific characters (i.e. the motifs), even if this specificity lies upon one position only.

To avoid cross-recruitment between enzyme or protein subclasses, OxyGene uses an anchor-driven annotation process. Each anchor is a "subclass identifier", corresponding to one or several conservation patterns likely to be responsible for specific functions. As mutations may result in the loss of biological function, we hypothesize that important amino acids are highly conserved across protein (sub)families. This functional and/or structural conservation is believed to be detectable as significant conserved residue patterns. Based on this assumption, we used, for each different ROS/RNS-scavenger subfamilies, a published set of functional enzymes and highly similar proteins (obtained by BLAST) to generate a significant number of representative sequences for each OxyDB. Using multiple alignments procedures, conserved or substitutive amino acid [42] patterns were chosen, in each set or subset of proteins, without discriminating between functional and non-functional regions. Each resulting anchor is composed of one or several motifs (regular expressions in PROSITE format), separated by spacers and organized as Boolean combinations without statistical scoring.

#### Supervised and iterative annotation

The anchor-based approach guarantees the absence of cross-recruitment between subclasses. However, to ensure that no anchor may falsely detect functionally unrelated proteins (false positives) or overlook a protein that carries out the function (false negatives), OxyGene uses an iterative and manually supervised (by human curators) process. Each anchor is exhaustively validated in non-redundant databases (see OxyGene annotator) and revisited every three months on new genomes to confirm the complete accuracy of OxyGene predictions.

### OxyGene Components (Figure 1)

**Figure 1:**
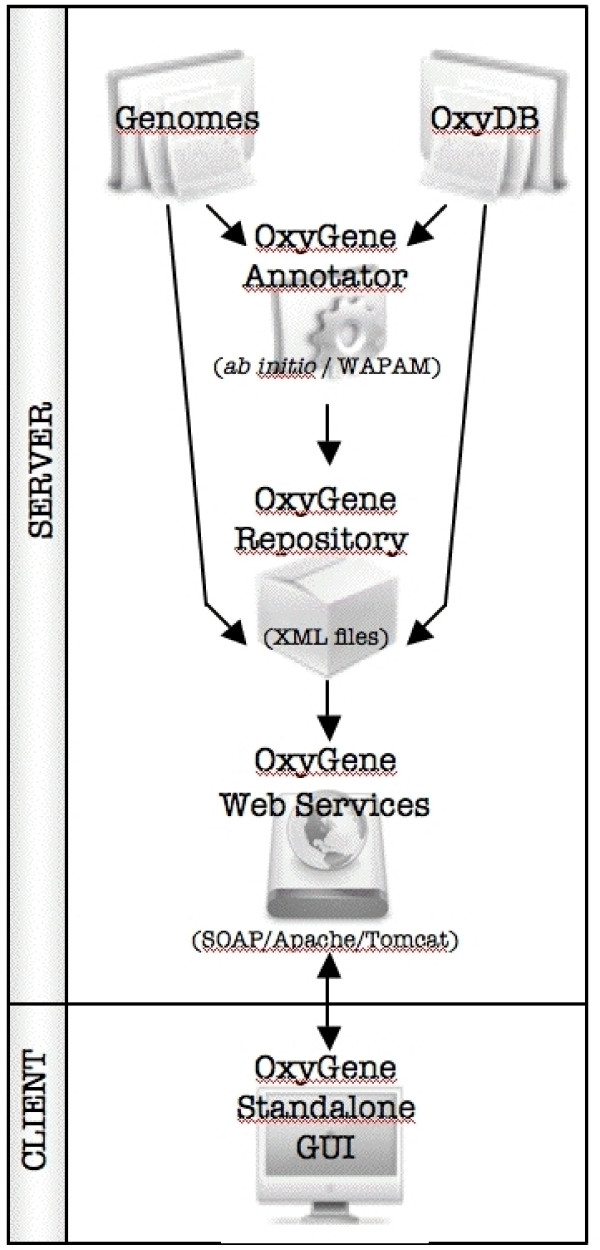
**Schematic workflow of the different components of OxyGene platform**. The OxyGene annotator inputs are NCBI whole genomes and OxyDB databases. Outputs are stored in the OxyGene XLM repository and are publicly accessible using the standalone OxyGene GUI through dedicated web services.

#### The OxyDB Database

OxyGene integrates an XML database incorporating new manually extracted information for each ROS/RNS enzyme and organized into seven fields of data (additional file 3):

1- The **name of the subsystem**: Although we have currently only implemented the detoxification subsystem, other subsystems, for example repair and reduction, are planned for inclusion.

2- The **OxyDB nomenclature**: This classification conforms to the IUBMB (International Union of Biochemistry and Molecular Biology) architecture and contains four levels: classes that correspond to the enzymatic activity (*e.g*. catalase) and three levels for subclass subdivisions defined using combinations of biological data from the literature and tree-based functional clustering (for details, see the classification table on our web page).

3- The **OxyDB anchors**: These are checked quarterly against all new (whole, being assembled and incomplete) genomes; patterns are refined if required.

4- The **OxyDB function confidence**: This rates the degree of confidence (DC) for each OxyDB_ID function and can be described as follows: DC_1 corresponds to experimentally demonstrated and published functions; DC_2 relates to an indirect function assertion (*e.g*. mutant, phenotype, microarrays or translational fusions) and DC_3 is based on sequence similarity to proteins rated DC_1 or DC_2.

5- The **OxyDB chemical reaction(s): **This "field" provides the main chemical reaction(s) catalyzed by each OxyDB_ID, as described in KEGG [43], SwissProt_Expasy [44] and MetaCyc [45].

6- The **corresponding EC number: **This allows each OxyDB_ID to be linked to the corresponding enzyme commission number(s) when available.

7- Additional descriptions. These details provide useful knowledge about each OxyDB_ID functions and publications.

#### The OxyGene annotator

The OxyGene annotator was developed in C++ and is embedded in a python script to generate the OxyGene precompiled data repository (see below). It performs an *ab initio *gene identification based on the new manually supervised OxyDB ontology (see above).

First, the OxyGene annotator identifies each motif that composes an anchor on six frames of translated DNA using a motif search tool called WAPAM [46] (Weighted Automaton Pattern Matching), available at Ouest-Genopole bioinformatics platform GenOuest [47]. All Bacteria and Archaea in the NCBI comprehensive genome database (at the time of this publication, 664 species) were parsed. This database is provided by GenOuest and updated monthly using BioMAJ biological database workflow engine.

All occurrences detected by WAPAM are filtered to satisfy (i) the inter-motif spacing and Boolean constraints of the anchor and (ii) the presence of a stop codon and the potential presence of start codons. At this point, all matching regions have been identified by OxyGene; only comparison with previous annotations needs to be performed. When perfect matches are found (same frame and stop position), the annotation start position is kept and its corresponding locus-tag and information are associated to the match. Sometimes the beginning of an anchor is found upstream of an annotated start; OxyGene then proposes a *re-annotated *tag. Loci that do not match any previously annotated genes are tagged as *de novo*. In the *de novo *and *re-annotated *cases, the longest ORF prediction is proposed. Finally OxyGene associates each locus with an evidence score (annotation score, AS): AS_1 for the experimentally validated protein defined by comparing genes found in a database of experimentally validated proteins [29,48]; AS_2 for proteins without biological evidence; and AS_3 for disrupted regions like frameshifts (when two separate motifs of the same anchor are found in two different frames of the same strand), or pseudogenes (one or two stops in frame). The procedure is repeated iteratively for each OxyDB_ID. All "*de novo*" and "re-annotated" loci are analysed by human curators and classified if needed into reannotated (alternative start) or frameshifted CDS, pseudogenes, and fragments (incomplete coding sequences).

Human curators verified all the OxyGene predictions using, for each OxyDB_ID, a systematic all-against-all NCBI blastp and tblastn verification with non-redundant (nr) databases for Bacteria (Taxid:2) and Archaea (Taxid:2157). This procedure constituted a quality control of the anchors with (i) refinement using detected false predictions (negative or positive) and (ii) validation of the motifs and Boolean combinations using incomplete genomes (Additional file 4).

#### The OxyGene repository and web services

The OxyGene repository stores the OxyGene annotator outputs indexed for each complete genome replicon. These files are XML-encoded (eXtensible Markup Language) and include every detected locus with its OxyDB_ID, annotation data (start and stop positions, frame), annotation type (frameshift, *de novo etc*), sequences (protein and nucleic), and function and evidence scores. This repository is updated incrementally.

The locally installed OxyGene Graphical User Interface (GUI) accesses the OxyGene repository through some private web services, implemented in Java 1.5 using the Apache AXIS 1.4 SOAP (Simple Object Architecture Protocol) library and deployed on the servlet engine Tomcat 5.5.20. The SOAP server provides a framework for exchanging XML data between the OxyGene repository and the GUI. The three web services are devoted to (i) initialization query that contacts OxyDB and genome databases, (ii) repertory query that allows a request for a genome, and (iii) OxyDB_ID query that retrieves all Bacteria or Archaea that contain at least one gene belonging to the requested OxyDB subclass.

## Utility and discussion

### The OxyGene Graphical User Interface (GUI)

The OxyGene platform has been developed as a client-server application. The server is installed at GenOuest Bio-Informatics platform [47]. The client is a Java application that needs to be downloaded locally by the users and which communicates with the server-side databases (OxyDB, OxyGene repository, Genomes data) through web-services. The client is platform-independent and runs with Java Run-time Environment version 5.0 or higher. The OxyGene GUI was successfully tested on Linux, Windows and Mac OS X.

**Figure 2:**
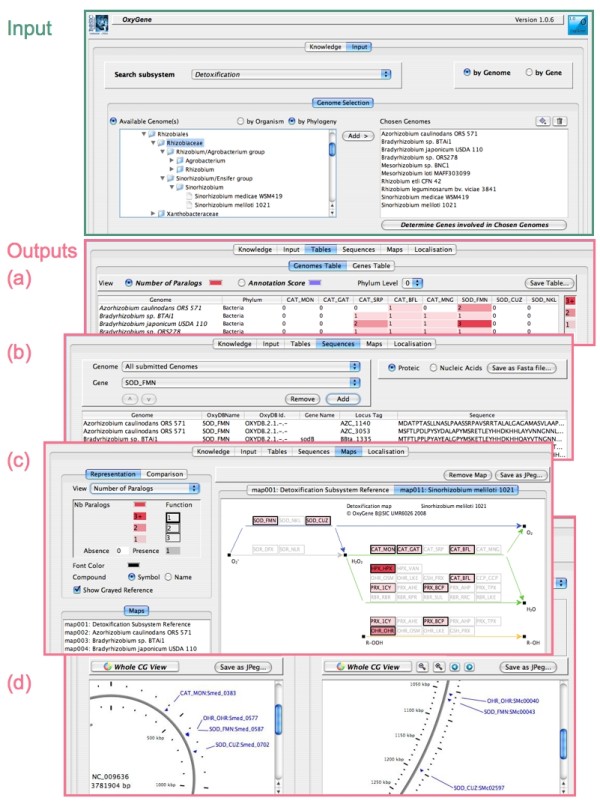
**The Graphical User Interface**. Some snapshots of the Graphical User Interface, showing the "Input" panel, where the genomes or OxyDB_ID are selected and submitted; the "Tables" panel, where the results are presented; the "Sequences" panel, from which files (in fasta format) of the desired sequences can be generated; the "Maps" panel, displaying the metabolic pathways involved in the subsystem; and finally the "Localisation" panel, where representations of genomic localisations can be viewed.

The client GUI, written using Java Swing API, is a unique window with six tabs entitled "Knowledge", "Input", "Genomes and Genes Tables", "Sequences", "Maps" and "Localisation" (Figure 2.). The "Knowledge" tab contains a summary of OxyGene *a priori *data (list of the available sequenced genomes, list of the OxyDB_IDs, OxyGene ontology and maps). The "Input" tab contains the query interface and supports two types of requests: by OxyDB_ID or by genome(s). The genomes can be selected by browsing an alphabetic list, by organism name completion or through a hierarchical taxonomic tree. Query results are accessible in tables and sequence tabs. The "Genomes Table" contains, for every 37 enzyme subclasses, the number of paralogs by genome together with their corresponding annotation confidence for each locus tag (Figure 2a). The "Genes Table" provide further detailed information such as locus tag, positions, frame, gene name and their links to NCBI [49] and KEGG [43]. The tables also discriminate between already annotated genes, re-annotated genes, *de novo *loci and also pseudogenes, and fragmented and/or shifted frames. These tables can be saved into tab-separated text files that are easily opened by spreadsheet applications. Additionally, nucleic and protein sequences can be selected and downloaded, in fasta format, in the "Sequences" tab (Figure 2b). The OxyGene "Maps" tab (Figure 2c) provides several options for visualizing and comparing the detoxification pathway of any sequenced genome on maps built using the jgraph.jar library [50]. A representation of the genomic localisation can be viewed, saved and compared in the "Localisation" tab (Figure 2d) which uses the CGView API [51]. A complete up-to-date "OxyGene GUI user guide" is available for download from the OxyGene website.

### Improvement of detoxification subsystem annotation

The OxyGene platform proposes a new classification that improves both the ontology and the annotation of the detoxification subsystem in whole prokaryotic genomes.

#### Classification and ontology

For example, by retrieving the original 956 NCBI descriptions of the five OxyGene catalase subclasses, we found 39 different functional assertions (Table 1). Some of these initial descriptions are (i) false (*e.g*. a DNA mismatch endonuclease and a putative chaperone protein in *Burkholderia pseudomallei*, and a phosphopyruvate hydratase in *Haemophilus influenzae*); (ii) inconsistent with the enzyme function (*e.g*. a monofunctional catalase annotated as a putative catalase/peroxidase in *Enterococcus faecalis V583*) or (iii) incomplete (*e.g*. HktE in *Pasteurella multocida subsp. multocida str. Pm70*, YdbD in *Bacillus licheniformis ATCC 14580*, YdhU in *Bacillus amyloliquefaciens FZB42*). Such diversity, heterogeneity and in some cases error in initial descriptions are found for all OxyGene detoxification classes; for example, there are more than sixty descriptions for the iron-manganese SOD_FMN.

**Table 1:** Comparison between the original NCBI descriptions and OxyGene new ontology

*NCBI versus OxyGene descriptions*	*CAT_BFL*	*CAT_MON*	*CAT_SRP*	*CAT_GAT*	*CAT_MNG*
catalase	25	251	26	76 5	27 21
hypothetical protein	6	14	7		
bi-functional catalase-peroxidase	3	-	-	-	-
catalase/hydroperoxidase HPI	146	-	-	-	-
catalase/hydroperoxidase HPII	-	6	-	54	-
catalase/(hydro)peroxidase KatG	9	-	-	-	-
catalase/peroxidase	55	1	-	-	-
catalase-peroxidase KatB	4	-	-	-	-
haem catalase	1	-	-	-	-
peroxidase/catalase (perA)	*2*	-	-	-	-
thr operon leader peptide	*1*	-	-	1	-
catalase KatA	-	12	-	-	-
catalase KatB or CatB	-	3	-	1	-
catalase KatE	-	1	-	1	-
catalase KatX	-	5	-	-	-
catalase precursor	-	8	-	-	-
HktE	-	1	-	-	-
major catalase in spores	-	1	-	-	-
monofunctional catalase	-	1	-	1	-
phosphopyruvate hydratase	-	1		-	-
putative catalase	-	3	-	-	5
vegetative catalase 1	-	2	-	-	-
catalase domain protein	-	1	24	-	-
catalase, protein srpA precursor	-	-	3	-	-
catalase-like	-	4	13	-	-
putative catalase	-	-	9	5	-
putative chaperone protein	-	-	1	-	-
catalase 2	-	-	-	1	-
catalase C	-	-	-	2	-
DNA mismatch endonuclease	-	-	-	-	1
manganese containing catalase	-	-	-	-	53
non-heme catalase KatN	-	-	-	-	2
pseudocatalase	-	-	-	-	2
spore coat protein (CotJC)	-	2	-	-	31
sporulation manganese (Mn) catalase	-	-	-	-	1
YdbD	-	-	-	-	2
YdhU	-	-	-	-	1
unknown protein encoded by prophage CP-933X	-	-	-	-	1

NCBI original descriptions of prokaryotic catalases and comparison with the new ontology proposed by OxyGene Numbers stand for *ab initio *annotated catalases that were re-classed by OxyGene.

OxyGene functional assertions satisfy the four core criteria of the definition of the ontology proposed by Gruber [52]: (i) Clarity in naming (*e.g*. CAT_MON is a typical monofunctional catalase); (ii) Coherence: no contradictions between function and description; (iii) Extendibility: new classes or subclasses can be added when necessary and (iv) Minimal ontological commitment: specifying the common term that defines all members of a subclass (*e.g*. CAT_MNG includes both spore- and non-spore catalases, so the term CAT_SPO was discarded).

#### Detection of mistakes in original annotations

**Figure 3:**
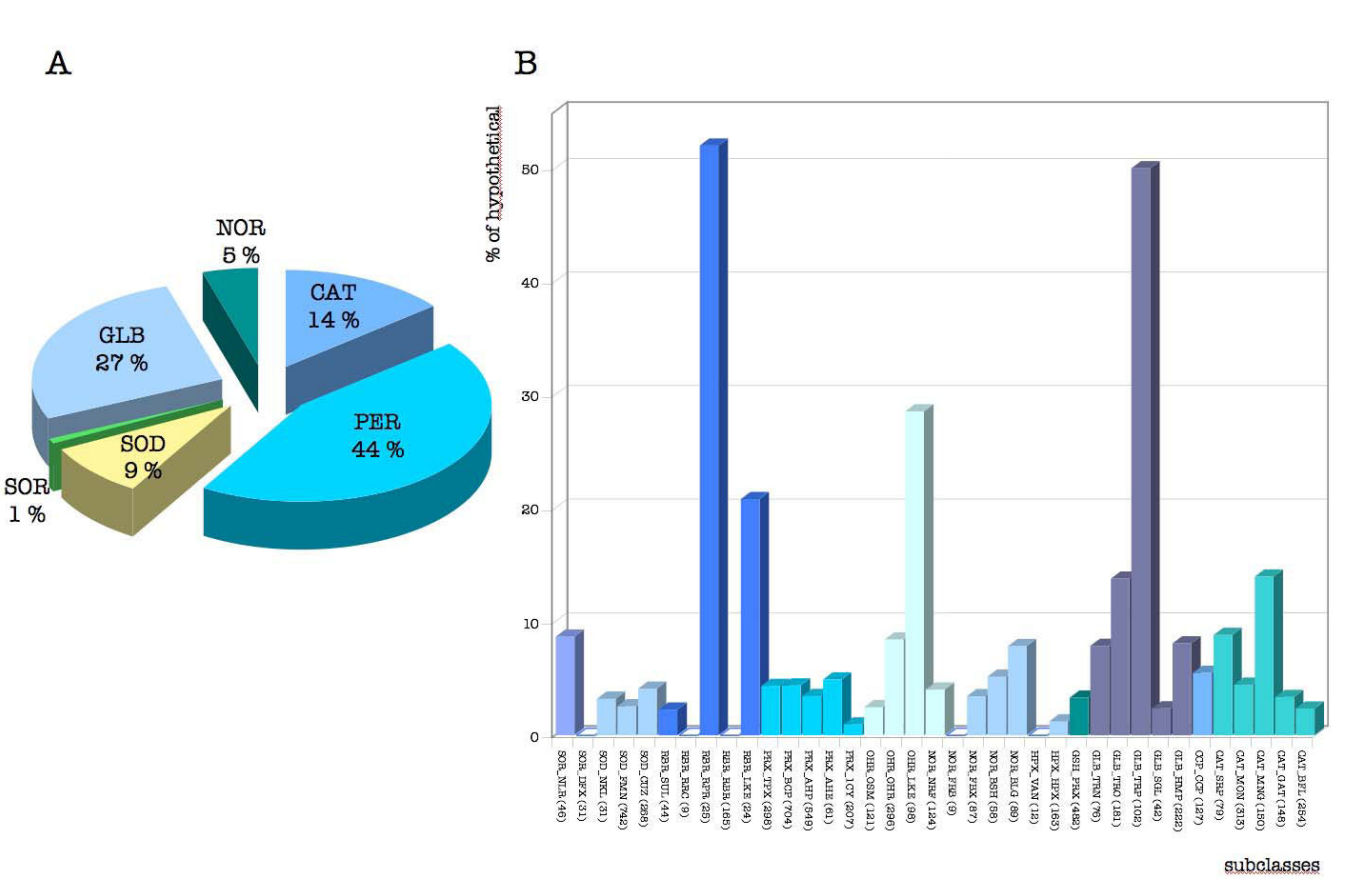
**Distribution of "hypothetical proteins" re-annotated by OxyGene among the various subclasses of detoxification enzymes**. A) Distribution of "hypothetical proteins" among classes: Hypothetical proteins re-classified as CAT (catalase), SOD (superoxide dismutase), SOR (superoxide reductase), GLB (nitric oxide dioxygenase), NOR (nitric oxide reductase) and as PER (peroxidase). B) Distribution of hypothetical proteins among subclasses: The graph represents the percentage of hypothetical proteins re-annotated by OxyGene for each enzyme subclass. Numbers in brackets are the total number of sequences encoding detoxification enzymes classified by OxyGene into each subclass.

Among the 6534 detoxification enzymes defined by OxyGene, 388 are annotated as "hypothetical protein" in NCBI files. Such "hypothetical proteins" are found in all classes with most (40%) in the peroxidase class, the other classes containing 1 to 14% (see Figure 3a). Regarding subclasses (Figure 3b), the presence of "hypothetical proteins" in recently described groups (GLB_xxx, [53]) can be explained; however, their presence in old, well-characterized enzyme subclasses, such as the catalase subclass, is more surprising [54]. We found that a BlastP analysis, using a "hypothetical" mis-annotated catalase as input, recruited other "hypothetical" mis-annotated catalases as first hits. This demonstrates how the absence of updating or correction in databases can lead to the propagation of annotation errors, as discussed by other authors [55,56].

At the scale of single genomes, the improvement in annotation of the detoxification subsystem by OxyGene is in some cases remarkable. For example, in *Vibrio harveyi ATCC BAA-1116*, OxyGene added four new detoxification proteins (PRX_AHP, PRX_BCP, OHR_LKE and SOD_CUZ), encoded on both chromosomes, raising the number of predicted ROS/RNS response genes from 12 to 16. Such omissions are observed in 165 complete genomes and this may have significant consequences for biological experiments (*e.g*. absence of phenotype in a mutant study) as well as on "*in silico*" studies (*i.e*. erroneous conclusions concerning detoxification abilities of organisms). Therefore, the OxyGene platform appears to be a powerful tool, and its impact on annotation will increase with the addition of new oxidative stress-related subsystems.

#### New annotations

Two indices may be used to assess the performance of the OxyGene platform: the specificity (improvements in original annotations, see above) and the sensitivity (discovery of missed features). Sensitivity is evidenced by the observation that OxyGene identifies 13 "overlooked" *loci*, all in intergenic regions (Additional file 5, table A). These new ORFs range from *ca*. 100 to 700 aa in length and can be identified from the presence of functional domains (catalases, superoxide dismutases etc). This high sensitivity is observed for all OxyDB classes and may be due to the combination of the *ab initio *and *anchor-driven *strategies used by OxyGene.

Additionally, OxyGene detected eight alternate translational starts sites (TSS), all predicted upstream from the originally annotated TSS (Additional file 5, Table B). All these reassignments of TSS were based on extensive comparative genomic analysis. In all cases, the amino acid homology could be significantly extended by between 30 and 92 residues. As TSS mis-annotations affect the prediction of protein function, location (signal peptide) and transcriptional regulation, it is essential to accurately re-annotate these loci in OxyGene.

OxyGene also detected seven new frameshifted genes, ten pseudogenes and four fragments (not shown). For each of these cases, it will be necessary to determine whether these "interruptions" are due to sequencing errors or to mutations events (insertion of transposable elements, point mutation). If confirmed, these OxyGene predictions would indicate that genes of the detoxification subsystem are subject to deleterious events.

### Characterization of detoxification subsystems

The OxyGene platform is the first tool that enables quick, reliable, and comparative (quantitative and/or qualitative) analysis of 664 prokaryotic detoxification subsystems.

#### Subsystem quantitative and qualitative diversity

**Figure 4:**
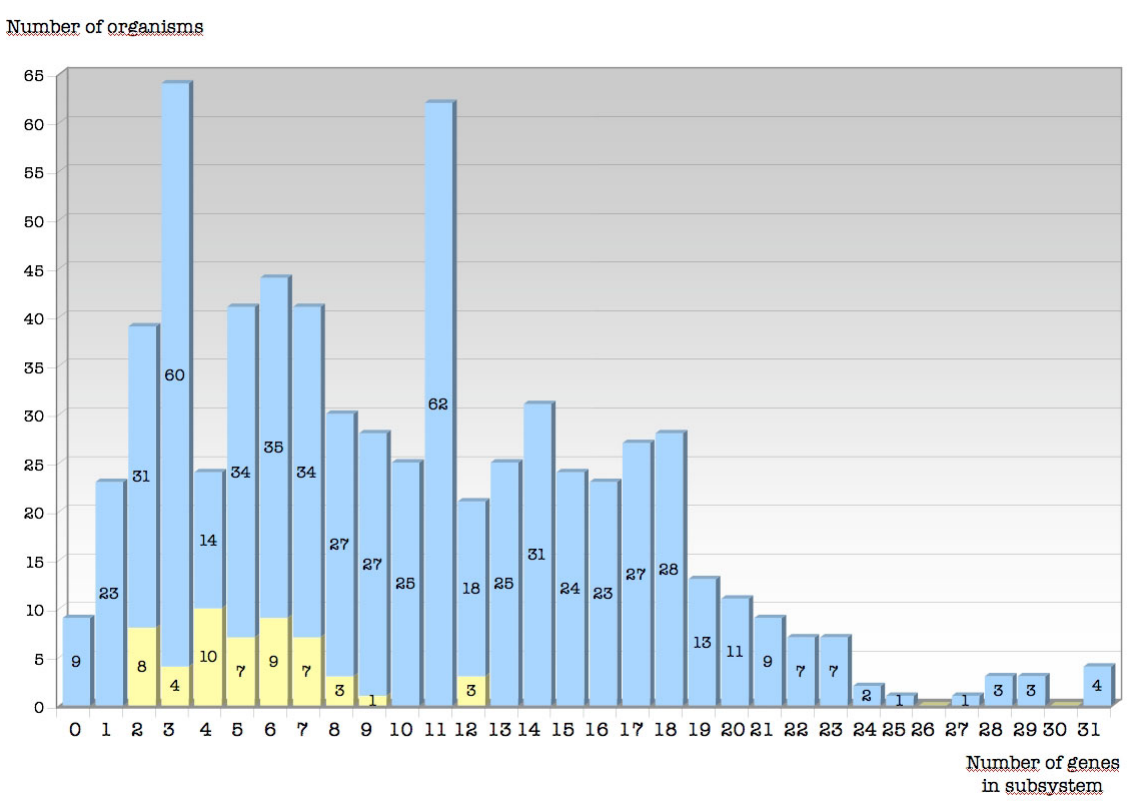
**Distribution of the detoxification genes in Bacteria and Archae**. Histogram showing the number of sequenced genomes of Archaea and Bacteria possessing any given number of detoxification genes.

No genome possesses all 37 OxyDB detoxification subclasses (Figure 4): there are between 0 and 31 detoxification genes in Bacteria species and between 2 and 12 in Archaea. The genomes with large numbers of detoxification genes present a high rate of paralogs (up to six OHR_OHR genes in *Burkolderia cenocepacia*), suggesting that no genome needs to contain all OxyDB classes. Of the nine genomes that do not possess any detoxification genes, three are endosymbiotic bacteria, but surprisingly six are free-living organisms (including *Lactobacillus *and *Brevibacterium*). This may suggest that: (i) some ROS/RNS response genes are still unidentified; (ii) other "satellite" subsystems (*e.g*. redox buffering, reduction, protein and DNA repair, etc.) serve as substitutives; (iii) there are compensatory processes in the environment, possibly involving cooperation between organisms.

In each of the six classes, the predominant subclasses are CAT_MON, SOD_FMN, PRX_BCP, GLB_HMP, NOR_NRF and SOR_NLR (Additional file 6). Genomes that contain only one or two detoxification genes tend to have a SOD and/or a peroxidase (SOD_FMN and PRX_BCP are present in 90% and 80% of the genomes, respectively). All OxyDB subclasses are found in Bacteria, but Archaea do not seem to use the whole spectrum of detoxification genes (16 subclasses are absent). Moreover, some OxyDB subclasses are differently distributed in the two kingdoms: for instance, the SOR_NLR, RBR_RBR and PRX_1CY genes, are much more frequent among Archaea than Bacteria, genomes whereas PRX_AHP, PRX_TPX and GSH_PRX genes are rare in Archaea but present in many Bacteria. Although the number of sequenced Archaea is still small (52 genomes), analyses of these differences will help determine the respective contributions of phylogeny inheritance, ecosystem adaptation and/or lifestyle in the selection pressures on the actors of the detoxification subsystem.

#### Detoxification subsystem comparative genomics

**Figure 5:**
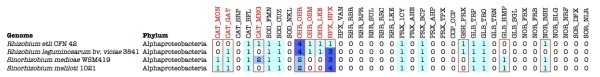
**Comparison of OxyGene results for four Rhizobiale genomes**. The table shows the number of genes present from each OxyDB subclass. The red boxes highlight quantitative differences.

The OxyGene tables can be used to compare several detoxification subsystems to help formulate biological hypotheses. For example, there are significant differences between four Rhizobiales (*Rhizobium etli*, *Rhizobium leguminosarum biovar viciae*, *Sinorhizobium meliloti *and *Sinorhizobium medicae*) despite them being closely related (Figure 5): some subclasses seem to be genus-dependent (CAT-MON and GLB-HPM in *Sinorhizobia*, GSH-PRX in *Rhizobia*) and others species-dependent (two CAT-MNG in *S. medicae *and none in *S. meliloti*) although the two species genomes are very similar (99.7% of identity of ribosomal RNAs [57]). The number of paralogs is also diverse, with one HPX-HPX in *R. etli *but more than three paralogs in the other three genomes.

The OxyGene detoxification maps are also very informative. As an example, the intersection of the four Rhizobiale maps shows that there are only nine common enzymes, mainly superoxide dismutase (SOD_FMN, SOD_CUZ) and peroxidase (PRX_1CY, PRX_BCP, HPX_HPX, CAT_BFL, OHR_OHR) activities (additional file 7). Both table and map representations can be completed by comparisons of the gene locations through the OxyGene CGview-based replicon viewer. Such treatment of *S. meliloti *and *S. medicae *replicons revealed that: (i) the detoxification genes seem to be randomly distributed and found on all replicons; (ii) some genomic regions are well-conserved (same genes, orientation, order, distance etc) whereas numerous genes are "singles" and in various locations; (iii) most of the syntenic regions contain the "core" Rhizobiale detoxification enzymes and (iv) additional loci are not necessarily correlated with additional replicons (additional file 8).

## Conclusion

The 21^st ^century is going to be a fruitful period with the start of the "genome" era. There are currently 3370 projects listed [58]: 813 published, 130 metagenomes, 2637 in progress (1801 Bacteria, 90 Archaea and 936 Eukaryotes). The forthcoming availability of 2500 bacterial genomes raises again the issue of annotation accuracy. Numerous problems need to be solved: omission of ORFs, partial or erroneous annotation, mistake propagation, absence of updating and disparity in function ontology. The most problematic consequence is the difficulty and even impossibility of efficiently exploiting the large and ever increasing amounts of "genomic" data. Therefore, it is important to design and develop dedicated bioinformatic tools devoted to supervised genomic data mining.

For this reason, we have developed OxyGene, an innovative platform that allows *ab initio *annotation and comparative analysis of detoxification subsystems in whole prokaryotic genomes. The annotation is manually supervised and supported by an iterative anchor-based process. The OxyGene GUI allows rapid and reliable identification of all genes encoding detoxification enzymes in complete genomes (even those that were previously not or mis-annotated), and then comparison of detoxification subsystems, maps and chromosomal locations. The accuracy of the predictions is maintained by regular human curator verifications.

OxyGene is unique. Indeed, no equivalent free software is currently available, and OxyGene is the first tool dedicated to oxidative stress. These ROS/RNS stresses are frequent in cells and the resulting imbalance between the generation and elimination of oxidants often leads to cell damage or death. Paradoxically, oxidative bursts are described as being essential signals for most prokaryote/eukaryote interactions. Consequently, we anticipate that OxyGene will make a very large contribution towards our understanding of the overall importance of detoxification systems.

In future development, OxyGene will include additional oxidative stress-related subsystems and connections with other metabolic pathways (e.g. on KEGG or METACYC). An "eukaryotic" version is in development.

## Availability and requirements

Home page: 

Operating systems: Mac OS × 1.4 and higher, Windows and Linux.

Programming languages: C++, Python and Java 5

Other requirements: Java JRE 5 (or higher) and Internet connection

Free for academic users. For use by non-academics: contact the B@SIC team.

## Authors' contributions

DT created the anchors, the web services, and the OxyDB repository and annotator. SA developed the GUI. CLM and AC supervised OxyGene results. FBH conceived and managed the project. All authors participated in data curation and in writing the manuscript.

## Supplementary Material

Additional file 1 **Classification of OxyGene subclasses.** Representation of an example (with the catalases) of the tree-based separation method used in OxyGene to classify enzymes. Click here for file

Additional file 2 **Comparison of BLAST, PSI-BLAST and HMMR capacity.** Comparison of BLAST, PSI-BLAST and HMMR capacity to recruit sequences belonging to a given class specifically. Click here for file

Additional file 3 **OxyGene XML initialisation file.** Sample of the information contained in the XML initialisation file. Click here for file

Additional file 4 **OxyGene anchor-based annotation.** The figure represents the schematic workflow of the anchor-based annotation validation process of OxyGene. Click here for file

Additional file 5 **Table with *re-annotated *and *de novo *loci detected by OxyGene.** The two tables provide details for reannotated and *de novo *detoxification loci detected by OxyGene. Click here for file

Additional file 6 **Detoxification subclasses distribution in complete genomes.** The two histograms show the number of prokaryotic sequences for each subclass, in Archaea and Bacteria. Click here for file

Additional file 7 **OxyGene detoxification maps.** The data provides a comparison of four Rhizobia detoxification maps. Click here for file

Additional file 8 **OxyGene synteny representation.** Comparison of oxidative gene locations in *S. meliloti *and *S. medicae *using the OxyGene CG viewer. Click here for file
