# Supplementary material for: OxyGene: an innovative platform for investigating oxidative-response genes in whole prokaryotic genomes
Source: BMC Genomics. 2008 Dec 31;9:637. doi: 10.1186/1471-2164-9-637 (PMC2631583; doi:10.1186/1471-2164-9-637)
Supplement: Additional file 1 — Classification of OxyGene subclasses. Representation of an example (with the catalases) of the tree-based separation method used in OxyGene to classify enzymes. [file 1471-2164-9-637-S1.pdf]

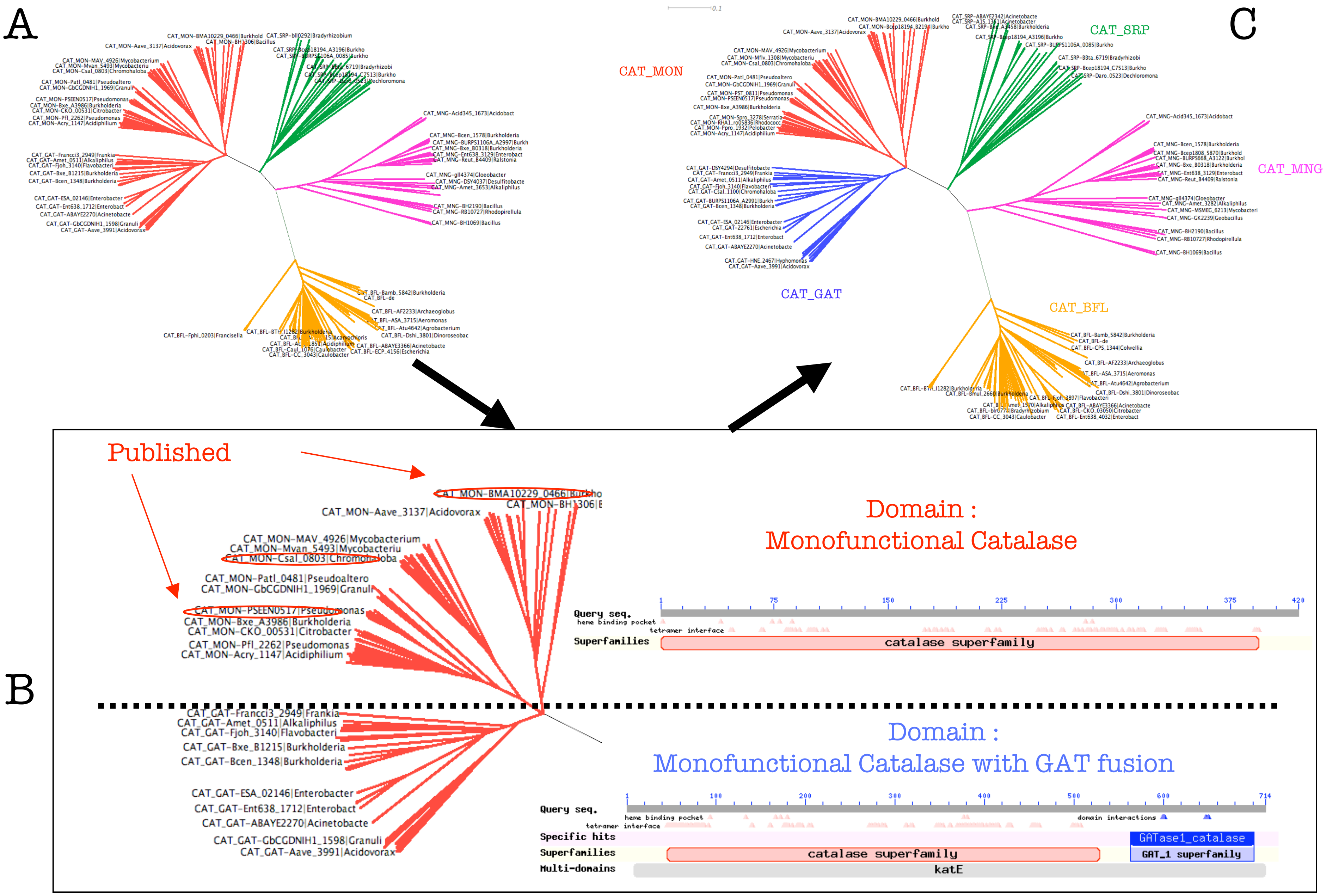

Using tree-based separation (Neighbour-Joining), 4 groups are observed for the catalase class (A). Complementary analysis exploiting data from the literature and domain conservation (B) allowed a classification refinement of the red branch (C) and thus the creation of 5 catalase subclasses (D).
