# Supplementary material for: OxyGene: an innovative platform for investigating oxidative-response genes in whole prokaryotic genomes
Source: BMC Genomics. 2008 Dec 31;9:637. doi: 10.1186/1471-2164-9-637 (PMC2631583; doi:10.1186/1471-2164-9-637)
Supplement: Additional file 2 — Comparison of BLAST, PSI-BLAST and HMMR capacity. Comparison of BLAST, PSI-BLAST and HMMR capacity to recruit sequences belonging to a given class specifically. [file 1471-2164-9-637-S2.pdf]

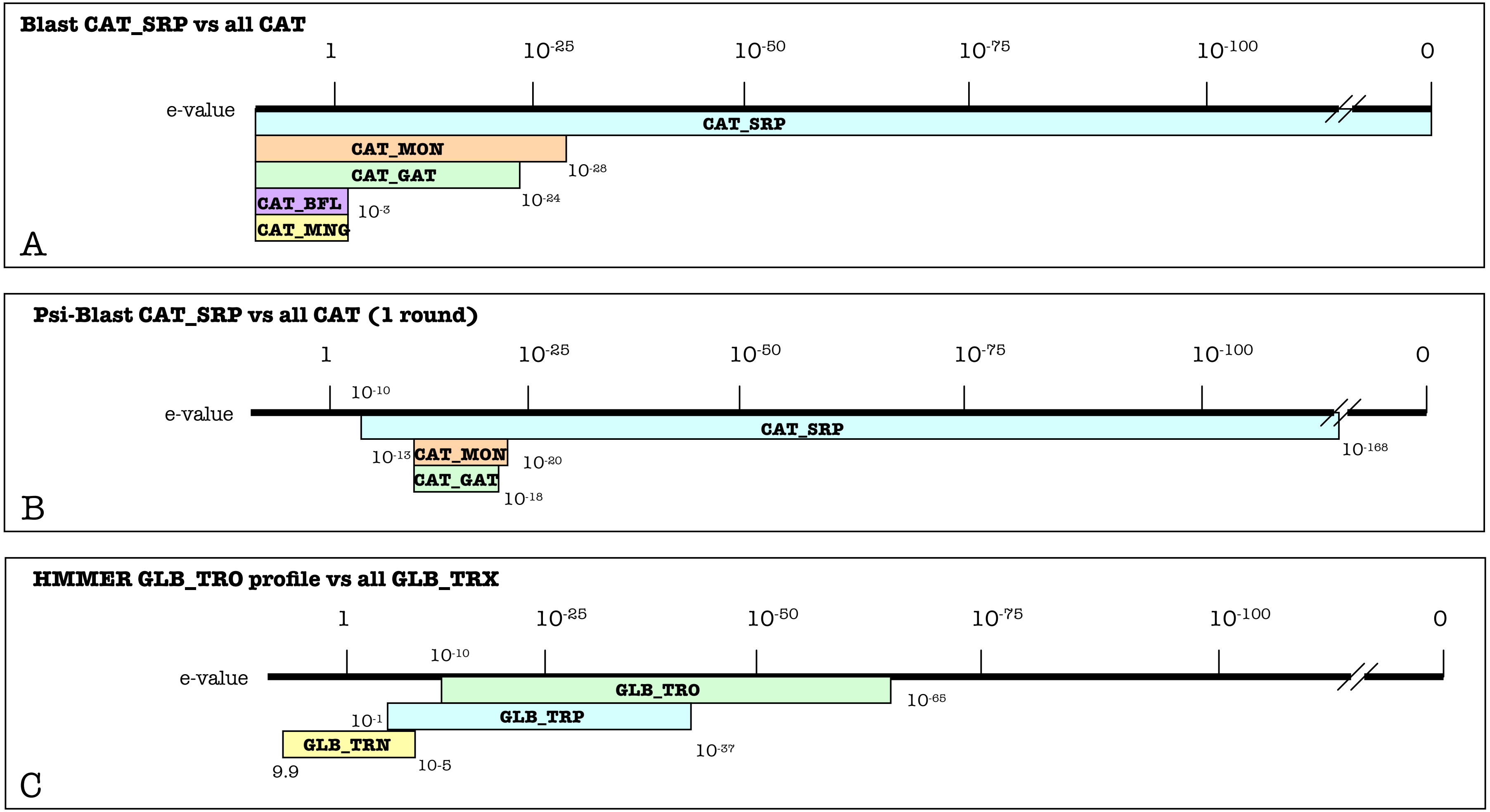

A: all sequences found by OxyGene belonging to the CAT_SRP class were blasted on a database containing all the catalases (haem, monofunctional, bifunctional and manganese catalase) found by OxyGene. The e-value range of hits for each OxyDB class was recorded.
B: one CAT_SRP sequence was PSI-blasted on a database containing all the catalases found by OxyGene. The e-value range of hits for each class in the first round was recorded.
C: an HMMER profile was obtained with an alignment of the GLB_TRO found by OxyGene. This profile was used to search a database with all the truncated globin sequences found by OxyGene. The e-value range of hits for each OxyDB class was recorded.
