# Supplementary material for: OxyGene: an innovative platform for investigating oxidative-response genes in whole prokaryotic genomes
Source: BMC Genomics. 2008 Dec 31;9:637. doi: 10.1186/1471-2164-9-637 (PMC2631583; doi:10.1186/1471-2164-9-637)
Supplement: Additional file 3 — OxyGene XML initialisation file. Sample of the information contained in the XML initialisation file. [file 1471-2164-9-637-S3.pdf]

```
<Subsystem type="Detoxification">

 <OxyDBClass1 oxyDBID="OXYDB.1.-.-.-" name="Catalase">
   <OxyDBClass2 oxyDBID="OXYDB.1.1.-.-" name="Monofunctionnal">
       <OxyDBClass3 oxyDBID="OXYDB.1.1.1.-" name="Catalase monofonctionnal typical" oxyDBTag="CAT_MON">

            <Signature>[RH]-G-[TFLW]-x(2)-[KR]-[FMLY]-Y-[TS]-x(14)-F-x(2)-[NRKQG]-[DE]-x(3)-F & x(280,305) &
                        ![FY]-x-[DNEQ]-[HYF]-[YFT]-[QH]-x(3)-[FY]-x(2)-S-x(4)-E </Signature>

            <Size>mean=499.0; sd=43.3;</Size>

            <FunctionConfidence> 1 </FunctionConfidence>

            <Reaction>2 H[2]O[2] -> 2 H[2]O + O[2]</Reaction>

            <ECNumber>1.11.1.6</ECNumber>

            <Description>
                   Enzymes of this class show catalatic activity: they detoxify two molecules of
                   hydrogen peroxide by producing two molecules of water and one of dioxygen [PM_ID:7670638].
                   The CAT_MON class belongs to the heme monofunctional catalase superfamily. All catalases
                   of this superfamily share the same mechanism for the degradation of H2O2 [PM_ID:14745498].
                   The CAT_MON catalase is composed of one complete monofonctional catalase domain only.
                   It is the typical Heme Catalase. This sub-family corresponds to the clade I and Clade III
                   of Klotz et al. classification [PM_ID:12777528]</Description>
```
