# Supplementary material for: OxyGene: an innovative platform for investigating oxidative-response genes in whole prokaryotic genomes
Source: BMC Genomics. 2008 Dec 31;9:637. doi: 10.1186/1471-2164-9-637 (PMC2631583; doi:10.1186/1471-2164-9-637)
Supplement: Additional file 4 — OxyGene anchor-based annotation. The figure represents the schematic workflow of the anchor-based annotation validation process of OxyGene. [file 1471-2164-9-637-S4.pdf]

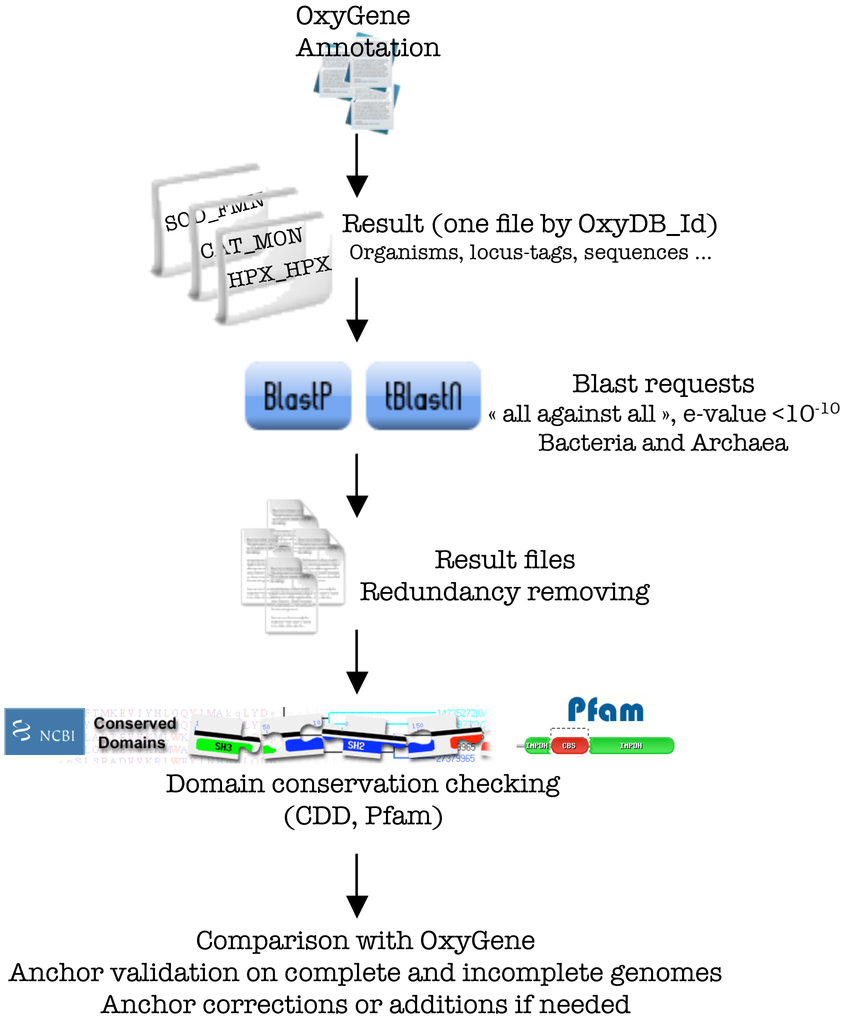

The OxyGene annotation outputs are retrieved by anchor (OxyDB_ID) and analysed one by one by human curators using homology (Blast) and domain conservation (CDD, Pfam) parameters before direct validation or correction.
