# Supplementary material for: OxyGene: an innovative platform for investigating oxidative-response genes in whole prokaryotic genomes
Source: BMC Genomics. 2008 Dec 31;9:637. doi: 10.1186/1471-2164-9-637 (PMC2631583; doi:10.1186/1471-2164-9-637)
Supplement: Additional file 5 — Table with re-annotated and de novo loci detected by OxyGene. The two tables provide details for reannotated and de novo detoxification loci detected by OxyGene. [file 1471-2164-9-637-S5.pdf]

Table A	Reannotated			
OxyDB	*Organism*	*Locus*	*New start*	*Some "long" orthologs*
GLB_HMP	*Acinetobacter baumannii* ATCC 17978	A1S_3085	30 aa (+)	ACIAD3226<br>PM8797T_10314<br>L8106_12750<br>BGP_2051
CAT_GAT	*Acinetobacter baumannii* ATCC 17978	A1S_1386	92 aa (+)	Pput_0132<br>PSPTO_5263<br>PFL_0074<br>Pfl_0064<br>Mfla_2377
HPX_HPX	*Acinetobacter baumannii* ATCC 17978	A1S_1833	71 aa (+)	ATEG_00342<br>BL01388<br>BlinB01000904
OHR_OHR	*Renibacterium salmoninarum* ATCC 3320[illegible]	RSal33209_0311	83 aa (+)	Mflv_5175<br>Haur_1741
RBR_RRC	*Campylobacter jejuni* 81-176	CJJ81176_0038	40 aa (+)	SUN_0438
RBR_RRC	*Campylobacter jejuni doylei* 269 97	JJD26997_0012	40 aa (+)	WS1374
RBR_RRC	*Campylobacter jejuni* RM1221	CJE0011	40 aa (+)	Cj8486_0011c
PRX_BCP	*Thermosynechococcus elongatus* BP-1	tsr0473	76 aa (+)	Ava_3881<br>alr3183<br>yc2152_c

Table B	*de novo*	
OxyDB	Organism	Position
CAT_BFL	*Escherichia coli* O157-H7 str. Sakai	76888-79098
PRX_BCP	*Brucella ovis* ATCC 25840	921820-921449
CAT_BFL	*Shigella dysenteriae* Sd197	3508064-3509647
CAT_GAT	*Bacillus licheniformis* ATCC 14580 (2)	4025950-4027407
GLB_TR1	*Burkholderia mallei* SAVP1	1855366-1855905
GLB_TR1	*Burkholderia mallei* ATCC 23344	1437471-143801[illegible]
NOR_BSH	*Brucella ovis* ATCC 25840	230904-232133
PRX_1CY	*Ralstonia eutropha* JMP134	874113-874646
PRX_AHP	*Shewanella oneidensis* MR-1	2878467-2877865
PRX_BCP	*Granulibacter bethesdensis* CGDNIH1	2193870-2194388
RBR_RPR	*Clostridium perfringens* SM101	2864011-2863472
SOD_FMN	*Nitrobacter hamburgensis* X14	2083982-2083587
SOR_NLR	*Nitratiruptor* sp. SB155-2	694853-694470
