# Supplementary material for: OxyGene: an innovative platform for investigating oxidative-response genes in whole prokaryotic genomes
Source: BMC Genomics. 2008 Dec 31;9:637. doi: 10.1186/1471-2164-9-637 (PMC2631583; doi:10.1186/1471-2164-9-637)
Supplement: Additional file 6 — Detoxification subclasses distribution in complete genomes. The two histograms show the number of prokaryotic sequences for each subclass, in Archaea and Bacteria. [file 1471-2164-9-637-S6.pdf]

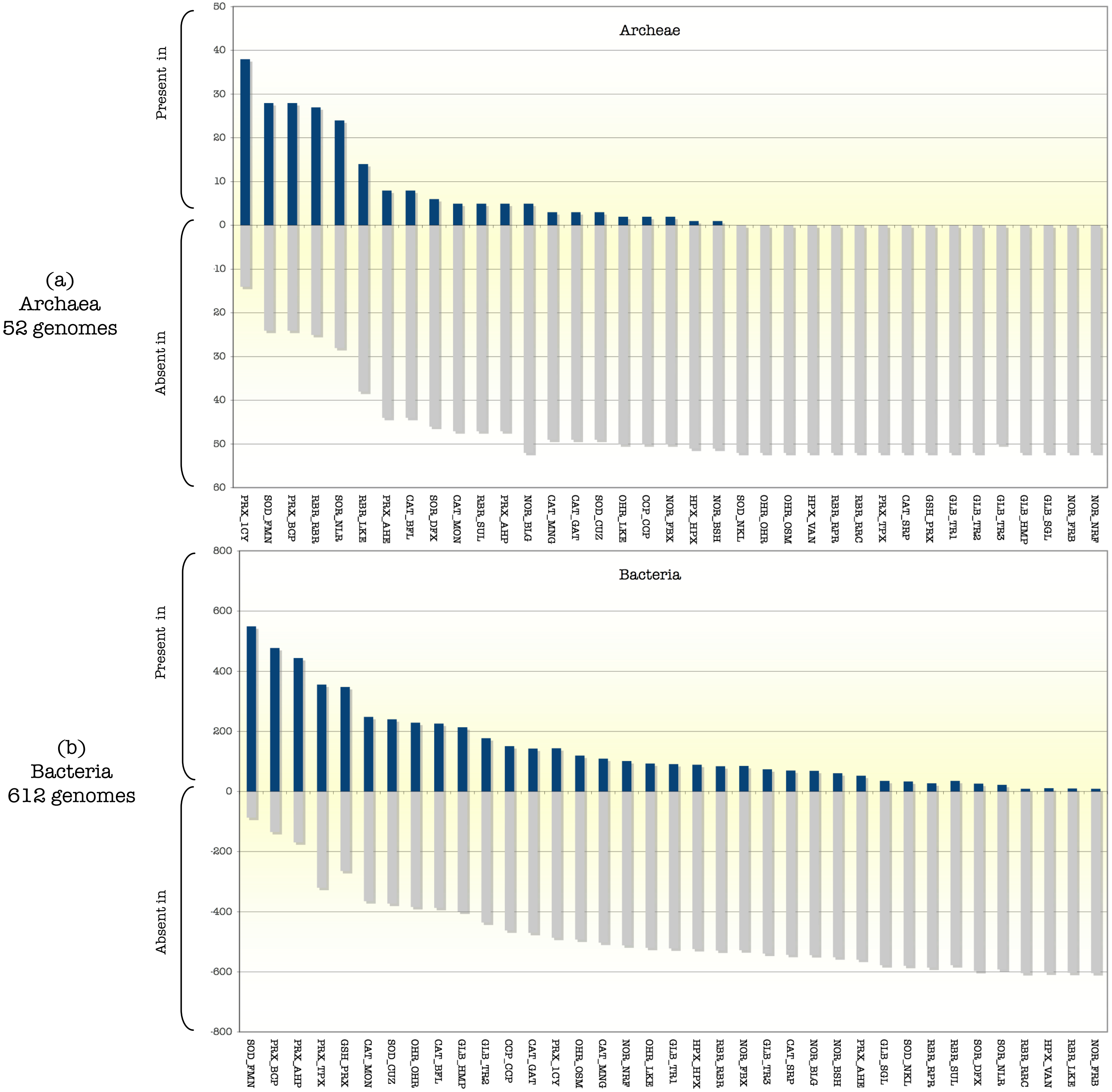

Genomes that contain (blue) or do not contain (gray) at least one gene of each particular detoxification subclass.
