# Supplementary material for: OxyGene: an innovative platform for investigating oxidative-response genes in whole prokaryotic genomes
Source: BMC Genomics. 2008 Dec 31;9:637. doi: 10.1186/1471-2164-9-637 (PMC2631583; doi:10.1186/1471-2164-9-637)
Supplement: Additional file 7 — OxyGene detoxification maps. The data provides a comparison of four Rhizobia detoxification maps. [file 1471-2164-9-637-S7.pdf]

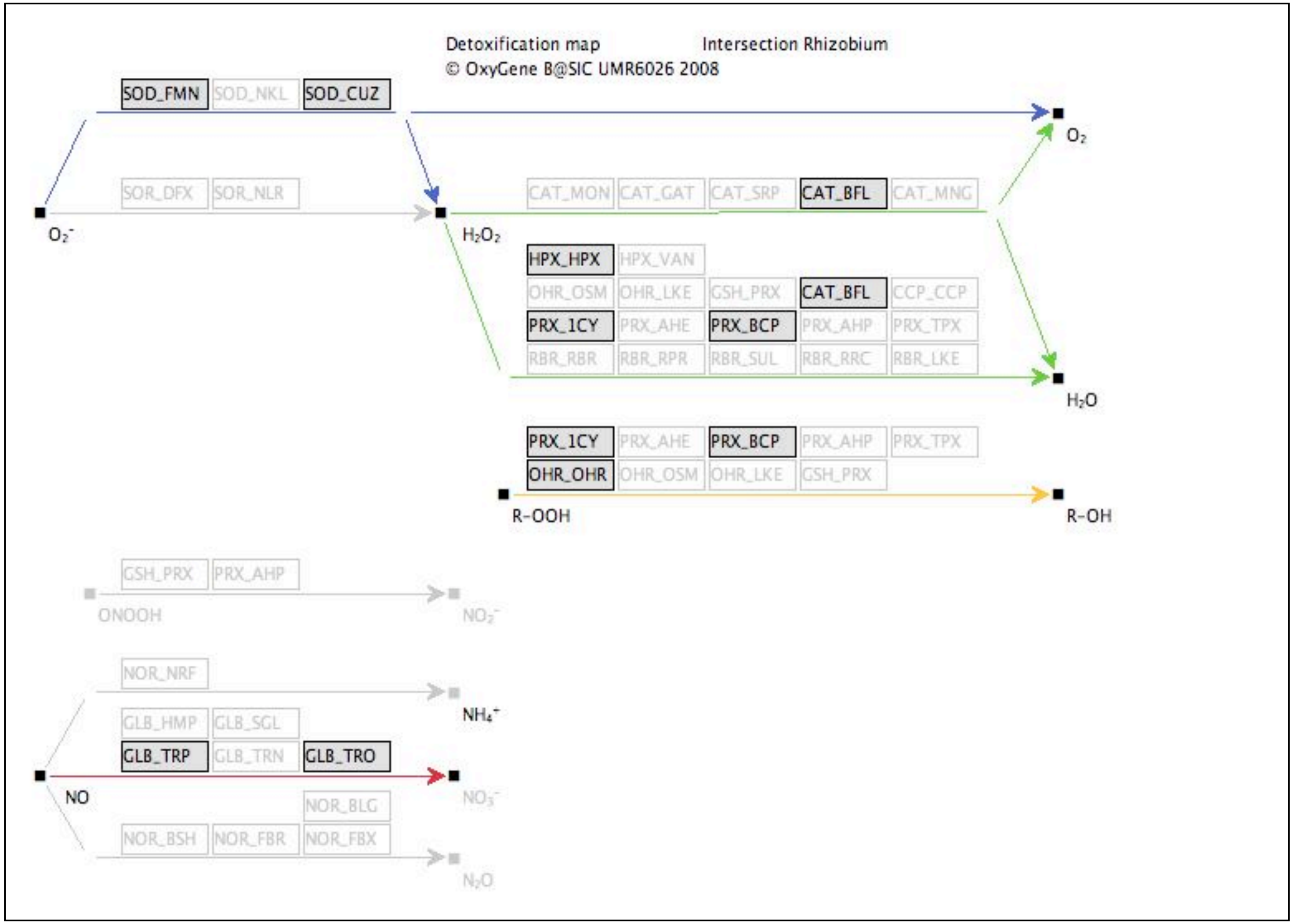

Intersection comparison map, provided by the OxyGene Map Tool, representing the nine common enzymes found in the four Rhizobia: Rhizobium etli strain CFN42, Rhizobium leguminosarum bv. Viciae strain 3841, Sinorhizobium medicae strain WSM419 and Sinorhizobium meliloti strain 1021.
