# Supplementary material for: OxyGene: an innovative platform for investigating oxidative-response genes in whole prokaryotic genomes
Source: BMC Genomics. 2008 Dec 31;9:637. doi: 10.1186/1471-2164-9-637 (PMC2631583; doi:10.1186/1471-2164-9-637)
Supplement: Additional file 8 — OxyGene synteny representation. Comparison of oxidative gene locations in S. meliloti and S. medicae using the OxyGene CG viewer. [file 1471-2164-9-637-S8.pdf]

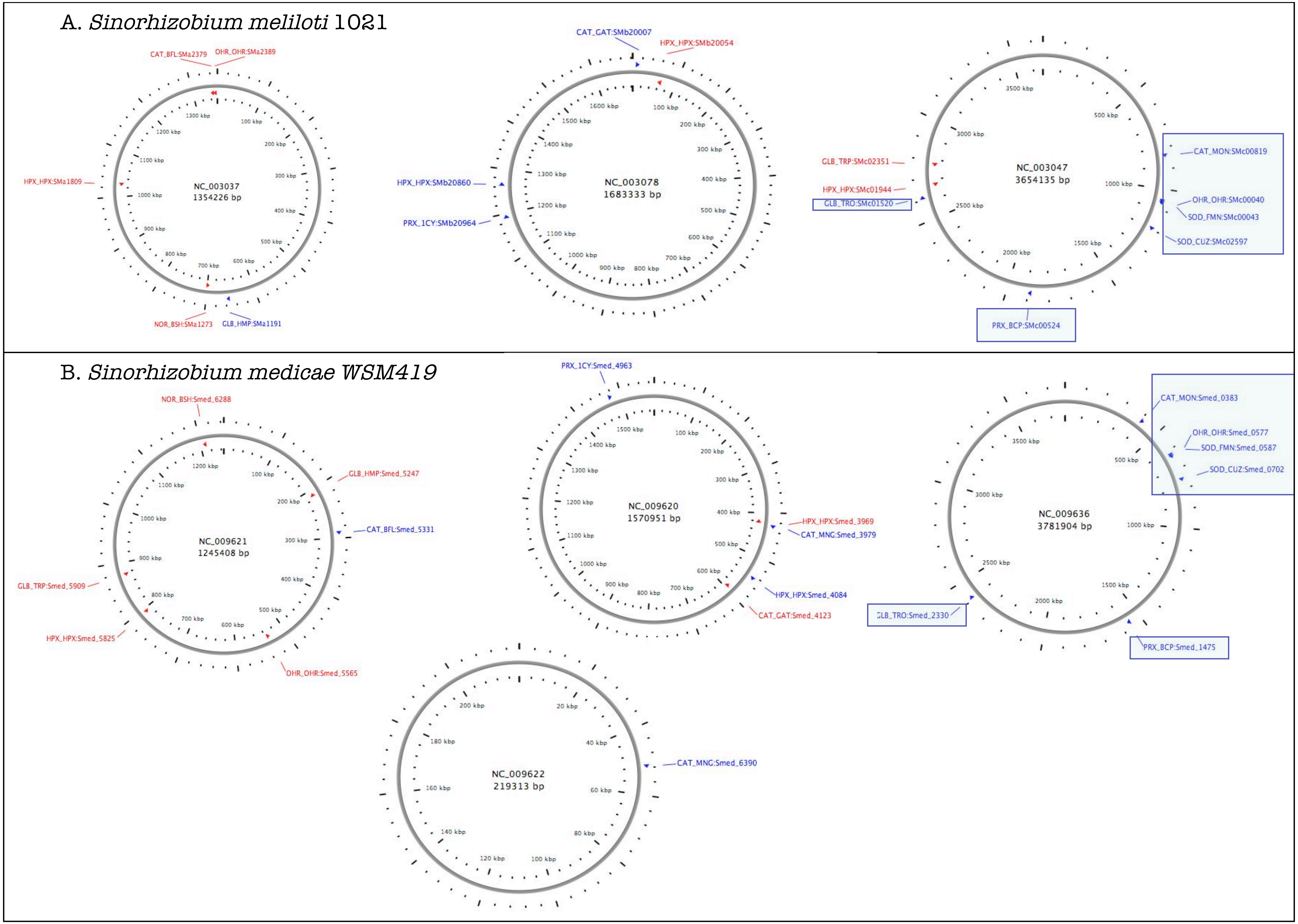

Physical location representations of detoxification subsystems reported by replicon using the OxyGene replicon map viewer for S. meliloti strain 1021 (A) and S. medicae strain WSM419 (B). The blue square indicates the conserved core genes.
